# Profiles of fungal metabolites including regulated mycotoxins in individual dried Turkish figs by LC-MS/MS

**DOI:** 10.1007/s12550-020-00398-5

**Published:** 2020-07-15

**Authors:** Michael Sulyok, Rudolf Krska, Hamide Senyuva

**Affiliations:** 1 Institute of Bioanalytics and Agro-Metabolomics, Department of Agrobiotechnology, IFA-Tulln, University of Natural Resources and Life Sciences, Vienna (BOKU), Konrad-Lorenz-Str. 20, 3430 Tulln, Austria; 2 Institute for Global Food Security, School of Biological Sciences, Queens University Belfast, University Road, Belfast, Northern Ireland BT7 1NN UK; 3 FoodLife International Ltd., ODTU Teknokent, 06800 Ankara, Turkey

**Keywords:** Dried figs, Fungal metabolites, Fluorescence, LC-MS/MS analysis, Aflatoxins, Kojic acid

## Abstract

Fungal metabolites including regulated mycotoxins were identified by a validated LC-MS/MS method in 180 individual Turkish dried figs from 2017 and 2018 harvests. Hand-selected dried figs were subjectively classified based on the extent of fluorescence. Forty-three fungal metabolites including eight EU-regulated mycotoxins were identified and quantified. Figs classified as being uncontaminated mostly did not contain aflatoxins above 1 μg/kg. Despite being “uncontaminated” from an aflatoxin perspective, kojic acid was present in significant quantities with a maximum level of 3750 mg/kg (0.375% w/w) and tenuazonic acid was also found (2 μg/kg to 298 mg/kg) in some figs. Notable in the screening of figs has been the presence of significant amounts of aflatoxin M_1_ (AFM1) in figs also containing significant levels of aflatoxin B_1_ (AFB1), which is the first time that AFM1 has been reported as naturally occurring in dried figs.

**Electronic supplementary material:**

The online version of this article (10.1007/s12550-020-00398-5) contains supplementary material, which is available to authorized users.

## Introduction

The production of dried figs employs some agricultural practices with a significant risk of fungal infection of the fruit and subsequent mycotoxin contamination. Figs ripen and shrivel on the tree and, after falling to the ground are collected daily, before sun-drying for 5 days or more (Gilbert and Senyuva 2008; Desa et al. 2019). During this period, a variety of different fungal species not least *Aspergillus flavus* and *Aspergillus parasiticus* can infect figs resulting in mycotoxin contamination. More than 30 years ago, aflatoxins were detected in a small number of individual dried figs from Turkey with extreme heterogeneity and an association between high aflatoxin levels in individual figs and bright greenish-yellow fluorescence on the surface, observable under UV light (Steiner et al. 1988). Even today, in Turkey, manual sorting of dried figs on a conveyor belt under UV light is employed as being the best option for identifying and removing aflatoxin-contaminated fruit. It is generally assumed (Steiner et al. 1988; Doster and Michailides 1998) that it is metabolites other than aflatoxins, such as kojic acid that are observed under UV light which provide an association with aflatoxin levels. In cotton, it has been shown that *A. flavus* produces kojic acid, which is then converted to the fluorescent compound by peroxidase in the plant (Marsh et al. 1969) which presumably also occurs in figs.

There have been a few studies focused on analyzing individual figs (Steiner et al. 1988; Şenyuva et al. 2008a) and only recent interest in the co-occurrence of mycotoxins in figs. Senyuva et al. (2005) reported the co-occurrence of aflatoxins with ochratoxin A (OTA) in dried figs and Karaca and Nas (2006) first reported patulin in aflatoxin-contaminated figs. A significant advancement in the identification of fungal metabolites has been achieved by the application of liquid chromatography–mass spectrometry (LC-MS). Applying LC-TOF-MS for mycotoxin analysis, about 50% of 52 individual figs were contaminated with OTA, aflatoxins B_1_, B_2_, G_1_, and G_2_; zearalenone, fumonisin B_2_ (FB2); and HT-2 toxin (Senyuva and Gilbert 2008). It should be noted that because of the high degree of heterogeneity of contamination of individual dried figs, mycotoxin levels reported in homogenized samples should not be directly compared with levels in individual figs.

Fusarium strains from moldy fig fruit collected in Italy were found to be capable of producing fusaric acid, beauvericin (BEA), fumonisin B_1_ (FB1), FB2, and fusaproliferin (Moretti et al. 2010). *Fusarium* is an agent causing endosepsis (internal rot) in fig fruit and it is a source of widespread infection in fig orchards in Turkey (Kosoglu et al. 2011) resulting in extensive contamination with FB1 and FB2. Cyclopiazonic acid (CPA) and β-nitropropionic acid (NPA) have also been found to be produced by *Aspergillus flavus* isolated from dried figs (Basegmez and Heperkan 2015). In a survey on *Alternaria* toxins in various foods, all fig samples were found to contain tenuazonic acid (TeA) at concentrations up to 1728 μg/kg (Lopez et al. 2016). Nine metabolites (AFB1, OTA, ochratoxin-α, kojic acid, emodin, altenuene, alternariol methyl ether, brevianamide F, and tryptophol) have been quantified at low levels in white and dark figs from Croatia (Petrić et al. 2018).

Although there is evidence of the widespread occurrence of fungal metabolites in figs, previous surveys have in each case focused on targeted groups of toxins. In contrast, in this paper, for the first time, we report the profiling by LC-MS/MS for approx. 295 mycotoxins and fungal metabolites in 160 individual figs which had been hand-sorted into different categories including visibly moldy figs. The objective has been to assess the effectiveness of the hand-sorting not only in terms of levels of aflatoxins in individual figs but also whether other mycotoxins of concern might be present, but not detected through UV screening. Additionally, this profiling has enabled a more detailed study to look for any association between levels of aflatoxins and other fungal metabolites particularly those exhibiting fluorescent characteristics.

## Materials and methods

### Procurement of samples of dried figs

Dried figs from the Aydin region of Turkey from 2017 and 2018 harvests after drying were selected, during manual sorting under UV light. Figs were categorized by the extent of bright greenish-yellow fluorescence. As well as highly contaminated figs, those classified as low or medium contamination would normally be rejected as part of the screening process. Separated individual figs were transported to Austria for analysis, chilled on receipt and during storage prior to analysis.

### Mycotoxin analysis

#### Chemicals and reagents

The chemicals and reagents used for the analysis were obtained from sources previously described (Sulyok et al. 2020). Standards for mycotoxin analysis were also prepared as described elsewhere (Malachova et al. 2018; Sulyok et al. 2020; Petrić et al. 2018). Briefly, 62 intermediate mixes prepared from stock solutions of analyte standards were mixed into one working solution, which was used for spiking and calibration.

#### Extraction

Individual figs were cut and pieces immersed in liquid nitrogen and subsequently ground with an Osterizer blender (Sunbeam Oster Household Products, Fort Lauderdale, FL, USA). The homogenized samples were weighed into 50-mL Falcon tubes and acetonitrile/water/acetic acid (79:20:1, v/v/v) was added for extraction at a ratio of 4 mL/g sample. Extraction was performed for 90 min at room temperature with a GFL 3017 rotary shaker set at 180 rpm (GFL, Burgwedel, Germany), and samples were allowed to precipitate. An aliquot (0.5 mL) of the extract was diluted with 0.5 mL of acetonitrile/water/acetic acid (20:79:1, v/v/v) in 1.5-mL vials and 5 μL of the diluted sample extract was directly injected into the LC–MS/MS system. Samples with levels of analytes exceeding the calibration range were re-analyzed after the respective extract being diluted appropriately.

#### LC-MS/MS

LC-MS/MS was conducted with a Sciex QTRAP® 5500 triple quadruple MS/MS (Sciex, Foster City, CA, USA) coupled with an Agilent 1290 binary UHPLC (Agilent Technologies, Waldbronn, Germany). The chromatographic conditions were strictly followed as described elsewhere (Sulyok et al. 2020).

#### Quantification

Calibration was based on external calibration using standards prepared in neat solvent. The results were corrected for apparent recoveries. Multiquant® 3.0.2 was used for data processing.

#### Method performance

Recoveries of the extraction step and matrix effects and determination of the method precision and within-laboratory reproducibility were determined by spiking experiments (Sulyok et al. 2020). The limits of detection (LOD) and quantification (LOQ) were determined following the EURACHEM guide (Magnuson and Örnemark 2014). All 12 results submitted for figs within a proficiency testing program organized by BIPEA (Genevilliers, France) were within the satisfactory range of − 2 < z < 2 (Sulyok et al. 2020).

## Results and discussion

### Method performance

The method performance for 43 metabolites in terms of recoveries and limits of detection (LOD) are provided in supporting information Table S1. Within-laboratory repeatability (*n* = 7) was found to range between 6.0 and 12.4% for aflatoxins in dried figs (Sulyok et al. 2020).

### Aflatoxins in figs from 2017 and 2018 harvests

A total of 180 individual figs from 2017 and 2018 harvests in Turkey were analyzed using the LC-MS/MS method capable of screening for a total of 700 mycotoxins and fungal metabolites, of which 43 different metabolites were identified. Where standards were available with the exception of nigragillin and aspulvinone E, all others were quantified. A summary of the results in terms of numbers of figs containing different metabolites and ranges of concentrations are shown in Table 1. This overview of the data does not provide insight into the differences in the patterns of metabolites in individual figs, and therefore, for a detailed examination, a full set of data for the 2017 and 2018 harvests is provided in supporting information Table S2. For simplicity, all data in this EXCEL dataset in Table S2 has been rounded to the nearest integer and shown as < 1 even if the actual LOD was below 1 (see Table S1). When LODs were > 1, the results are reported as < or > the numerical LOD. All results were corrected for the apparent recovery obtained from spiking blank figs with a mixture of standards.

**Table 1:** Summary of the numbers of individual figs contaminated with different mycotoxins and the range of concentrations from both 2017 and 2018 harvest years

Mycotoxins	Numbers of figs and levels in μg/kg (mg/kg for kojic acid)					
	2017 harvest year			2018 harvest year		
	< 1	> 1	Range of levels	< 1	> 1	Range of levels
AFB1	49	31	1–4320	36	64	2–22300
AFG1	66	14	1–6630	37	63	1–24600
AFM1	62	18	3–427	37	53	1–2730
Aflatoxicol	66	14	1–114	79	21	2–818
BEA	50	30	1–1010	98	2	6–42
Me-ST	62	18	3–747	50	50	1–2020
NPA	45	35	4–16000	44	56	13–26300
OTA	72	8	1–11400	84	16	5–77300
OTB	74	6	2–2230	92	8	5–3100
ST	61	19	1–3910	55	45	1–135
	**<LOD**	**>LOD**	**Range of levels**	**<LOD**	**>LOD**	**Range of levels**
Bikaverin	65	15	4–41	96	4	5–137
CPA	67	13	38–1470	88	12	109–8880
FB1	74	6	30–1430	96	4	13–335
FB2	75	5	7–130	99	1	69
TeA	18	62	18–2940	37	63	45–299000
Kojic acid	1	79	0.061–98	0	100	0.21–3750

For individual figs, the samples for 2017 were coded based on expert manual screening under UV light with U indicating uncontaminated, L = low contamination, M = medium contamination, and I = intense fluorescence and therefore assumed to be highly contaminated. All contaminated figs showed evidence of bright greenish-yellow fluorescence on the surface, but it was a subjective decision as to whether this constituted L, M, or I contamination. For 2018 harvest, only figs classified as U or I were selected for analysis. Each fig has its own unique identification number.

From Table S2, it can be seen that all 20 individual 2017 figs which were assessed as being uncontaminated contained no detectable aflatoxins, as was the case with 17 out of 20 figs which were assessed as having low contamination. For these 17 figs, there was clearly some background fluorescence visually detectable, but not associated with the presence of aflatoxins. One fig (sample L-07-17) appears to have been misclassified as having low contamination, as it was found to contain roughly equal levels of AFB1 and AFG1 of much the same order of magnitude as those generally found in the medium contamination group. Twelve out of 20 figs classified as having a medium level of contamination were completely uncontaminated, again indicating that visual fluorescence is not always associated with aflatoxin contamination. Of the 20 figs that exhibited intense fluorescence, 11 figs were essentially uncontaminated (levels of both AFB1 and AFG1 below 6 μg/kg) again indicating that fluorescence was not exclusively an indicator of high levels of aflatoxins. Overall, from Table S2, it can be seen that for all categories of contaminated figs, levels of AFB1 ranged from 64 to 6220 μg/kg and levels of AFG1 ranged from 80 to 6630 μg/kg. Eight contaminated figs had levels of AFG1 ranging from about half of the level of AFB1 to levels exceeding that of AFB1 (e.g., fig number I-08-17 with AFG1 level 50% higher than AFB1). This pattern is indicative of *A. parasiticus* infection whereas, for the other 12 contaminated figs, levels of AFG1 were below the LOD in most cases, despite AFB1 levels ranging from 172 to 2980 μg/kg this pattern being indicative of *A. flavus* infection.

In Table S2, the results are presented for the analysis of 100 individual figs from the 2018 harvest of which 35 were classified as uncontaminated and 65 were found to have intense fluorescence. As with the 2017 harvest, none of the 35 figs classified as uncontaminated contained detectable aflatoxins (there was no low or medium classification for the 2018 harvest). Of the 65 intensely fluorescent figs, only six (9%) had levels of both AFB1 and AFG1 below 6 μg/kg compared with 55% of intensely fluorescent figs in the 2017 harvest although with a smaller sample size. There is therefore a distinct difference between 2017 and 2018 in the extent of misclassification based on fluorescence. Overall for 2018, it can be seen that for intensely fluorescent figs, levels of AFB1 ranged from 2 to 22,300 μg/kg and levels of AFG1 ranged from 1 to 24,600 μg kg^-1^. These high levels are consistent with reports of analysis of 62 Turkish dried figs from the 1985 harvest, where 11 figs contained AFB1 in the range 1000–10,000 μg/kg and one sample had levels exceeding 10,000 μg/kg (Steiner et al. 1988). However, for 50 individual figs from the 2005 harvest (Şenyuva et al. 2008a) rejected for human consumption, levels of AFB1 and AFG1 ranged from < 0.1 to 2201 μg/kg and < 0.1 to 734 μg/kg, respectively. These year-to-year differences are unsurprising as infection rates are influenced by a number of factors not least climatic conditions including drought (Bircan et al. 2008). From Table S2, it can be seen that for the 2018 harvest, 18% of figs had high levels of AFG1 being indicative of *A. parasiticus* infection, compared with 42% of those figs exhibiting a similar pattern in 2017. Comparably high levels of AFG1 in individual figs were also reported in a 1985 survey (Steiner et al. 1988).

One of the really surprising findings (see Table 1) has been the presence of AFM1 at levels ranging from 1 to 2730 μg/kg and levels of aflatoxicol ranging from 2 to 818 μg/kg. AFM1 is well-recognized as a hydroxylated metabolite of AFB1 excreted in milk and urine after the ingestion of AFB1 contaminated feed or food by animals or humans (Ketney et al. 2017; Frazzoli et al. 2016). However, these two metabolites are not commonly reported to be found in crops naturally contaminated with AFB1, such as cereals, nuts, and dried fruit and are reported here for the first time as being present in dried figs. As a proportion of AFB1, levels of AFM1 ranged from 1.7 to 9.8% for the 2017 harvest, whereas a significantly higher proportion of AFM1 was found in 2018 ranging up to 36% (e.g., fig number I-24-18). It has been suggested that most strong fungal AFB1 producers produce AFM1 as well at a ratio of approx. 1:100 (Jens Frisvad, personal communication) and also it is relevant that under culture condition, several fungi including non-toxigenic A *flavus* are capable of hydroxylation of AFB1 to AFM1 (Nakazato et al. 1990, 1991).

### Mycotoxins and metabolites other than aflatoxins

Table 1 shows the results for some selected mycotoxins found in individual figs from the 2017 and 2018 harvests for which a full data set can be found in supporting information Table S2. In addition to aflatoxins, these twelve other metabolites have been selected in terms of having been reported more widely in other matrices and were also found to be present in multiple figs in different categories.

3-Nitropropionic acid (NPA) originally reported to be produced by a strain of *A. flavus* (Bush et al. 1951) was found in 43% of 2017 harvest figs at levels ranging from 4 to 16,000 μg/kg and in 70% of the 2018 harvest ranging from 13 to 26,300 μg/kg. NPA has previously been produced under culture conditions from *A. flavus* species isolated from dried figs (Basegmez and Heperkan 2015), but has not been reported as occurring in figs themselves. In all cases, NPA was detected in figs which showed fluorescence (medium or intense) in some cases also associated with high AFB1 levels, but also in figs where no aflatoxins were present (e.g., figs from 2017 harvest containing 988 μg/kg, 9500 μg/kg, and 16,000 μg/kg of NPA, but containing < 1 to 2 μg/kg of AFB1). This demonstrates a clear advantage in categorizing fluorescent figs as unsuitable for human consumption even if AFB1 is absent, as NPA is a mycotoxin that has been confirmed to pose a health risk to humans, potentially causing central nervous system dysfunction and brain damage albeit not regulated (Matumba et al. 2017).

The presence of sterigmatocystin (ST) and *O*-methyl sterigmatocystin (Me-ST) which are both mycotoxins which are found as metabolic intermediates of aflatoxins (Sweeney and Dobson 1999) is unsurprising. ST was found in 23% of figs from the 2017 harvest at levels ranging from 1 to 3910 μg/kg and Me-ST in 35% of figs at levels ranging from 3 to 747 μg/kg. For the 2018 harvest, ST was found in 45% of figs at levels ranging from 1 to 135 μg/kg and Me-ST in 48% of figs at levels ranging from 1 to 2020 μg/kg. In all figs where ST or Me-ST was present, as expected, various levels of AFB1 were also found, with the exception of one intensely fluorescent fig (I-03-17) containing 3910 μg/kg of ST, but only 1 μg/kg of AFB1. MeST has previously been reported as occurring in dried figs (Senyuva et al. 2008b), but to our knowledge, this is the first report of the natural occurrence of ST which is otherwise commonly found in cereals and derived products as well as cheese. ST has been classified as a group 2B possible human carcinogen (IARC 1987), so there is obvious concern about its presence in food and feed.

Ochratoxin A (OTA) was only found in 8 of the 80 dried fig samples from the 2017 harvest, at low μg/kg levels in two samples but at 59 μg/kg, 72 μg/kg, 9150 μg/kg, and 11,400 μg/kg in the other samples. Similarly, OTA was only found in 16% of the dried fig samples from the 2018 harvest at levels ranging from 5 to 77,300 μg/kg. For 2017 harvest figs, in OTA-positive samples, OTB and OT-α were also detected at 5–44% and 1–4% of OTA levels respectively. All OTA-contaminated figs were found in the low or medium category of fluorescence in 2017, but none of the six figs contained detectable aflatoxin B_1_. The natural occurrence of OTA in dried figs has previously been reported (Heperkan et al. 2012; Di Sanzo et al. 2018; Senyuva et al. 2005; Senyuva and Gilbert 2008). Although OTA is not regulated in the EU in dried figs, a maximum limit of 10 μg/kg applies to dried vine fruit (EC 2006b) and therefore, by extrapolation, levels of OTA from 59 to 11,400 μg/kg are clearly unacceptable, notwithstanding the dilution implicit in application of the appropriate sampling plan (EC 2006a). Again as was the case with NPA, despite the absence of aflatoxin contamination, the removal of figs showing fluorescence leads to discarding of OTA contaminated figs with the fluorescence being indicative of mold activity rather than specifically of the presence of aflatoxins.

Tenuazonic acid (TeA) was found in 79% of the 2017 dried figs, and in 78% of the 2018 dried figs contaminated overall at levels from 18 to 299,000 μg/kg with a higher proportion of figs with more significant levels of TeA in 2018 than in 2017. Additionally, some other *Alternaria* metabolites were detected albeit at a low incidence and relatively low levels. Recently, TeA has been reported in 100% of 14 samples of dried figs (Lopez et al. 2016) at levels of 41–1730 μg/kg, although in tomatoes and tomato products, TeA is more widely recognized as being a significant mycotoxin contaminant.

Fumonisin B_1_ (FB1) was only found in 6 out of 80 individual figs in 2017 and in 4 out of 100 individual figs in 2018 crop at overall levels for the two years ranging from 13 to 1430 μg/kg. Generally, there was co-occurrence of FB2, FB3, and in some cases FB4. FB1 has previously been reported in dried figs (Kosoglu et al. 2011; Heperkan et al. 2012) as has FB2 (Senyuva and Gilbert 2008). In comparison with say corn which is susceptible to fumonisin contamination, the amounts found in dried figs are less significant. *Fusarium* is known to be responsible for causing endosepsis (internal rot) in fig fruits in Turkey (Kosoglu et al. 2011) and Italy (Moretti et al. 2010). Therefore, fumonisins can be regarded as pre-harvest contaminants of figs and while co-occurrence with aflatoxins and other mycotoxins might occur, it is not inevitable. In the EU, the maximum limit for fumonisins (FB1 + FB2) is 1000 μg/kg in maize intended for direct human consumption (EC 2006b).

Beauvericin (BEA) was found in 36% of the 2017 figs but only in 2% of the 2018 figs BEA is one of the so-called emerging *Fusarium* mycotoxins (Jajic et al. 2019) which has been reported predominantly as occurring in cereals and was previously found in dried figs (Di Sanzo et al. 2018). As an example of one of the more obscure fungal metabolites, levels of bikaverin were found in 19% of figs from 2017 but in only 3% figs from 2018. Bikaverin is a reddish pigment produced by different fungal species, most of them from the genus *Fusarium* (Limón et al. 2010) and has not been previously reported in dried figs although probably not specifically sought.

Kojic acid is notable with respect to the large quantities that have been found in dried figs with increasing levels consistent with the extent of fungal contamination. Kojic acid is an organic acid and is a secondary metabolite produced by several species of *Aspergillus* such as *A. oryzae*, *A. tamarri*, *A. parasiticus*, and *A. flavus* (Rosfarizan et al. 2010). The quantification of kojic acid eluting as a broad peak early in the ion chromatogram was achieved in LC-MS/MS although the sensitivity was poor (LOD = 20 μg/kg) but amounts present were very high. Kojic acid was detected in all individual figs with levels of a few hundred μg/kg in figs categorized as being uncontaminated or with low contamination through to intensely fluorescent figs having substantial levels with a maximum of 3750 mg/kg in one fig (number I-71-18) equating to 0.375% kojic acid by weight. The full data set for levels of kojic acid can be found in Table S2. The bright greenish-yellow fluorescence on the surface of dried has been suggested to be due both to kojic acid and to a fluorescent compound produced by peroxidase in the plant (Doster and Michailides 1998; Hruska et al. 2014). Therefore, fungi other than those which are aflatoxigenic which produce kojic acid may also show fluorescence. The fact that fluorescence is not exclusively correlated with aflatoxins has the benefit from a food safety perspective that figs which do not contain significant aflatoxin B_1_, but do contain other toxins such as NPA, OTA, or TeA, would nevertheless still be rejected after hand-sorting under UV light.

Nigragillin, previously found to be produced by fungal cultures isolated from dried figs (Senyuva and Gilbert 2008), is worthy of comment as a metabolite produced by *Aspergillus niger* which appears to be present in significant quantities in nearly all dried figs even in the uncontaminated category. Although no analytical standard was available for nigragillin, even without a standard for quantification, the very large peak areas are evidence of substantial amounts of this metabolite being present. Nigragillin has insecticidal activity (Isogai et al. 1975) and is therefore of interest in terms of overall profiling of fungal metabolites in dried figs.

Although there are many other publications reporting the occurrence of mycotoxins in dried figs, they mostly have undertaken target analysis for a limited number of mycotoxins and not therefore looked at the overall picture including the presence of other metabolites such as kojic acid. The results we report confirm the effectiveness of screening figs under UV light to remove those showing bright greenish-yellow fluorescence on the surface which mostly contain aflatoxins. However, the results also show that there are certain figs, which do not contain detectable aflatoxins, but nevertheless do contain other mycotoxins at significant levels. The screening therefore has the added benefit of identifying and removing from the food chain, those figs containing NPA, OTA, fumonisins, and TeA in addition to those containing AFB1.

## Electronic supplementary material

ESM 1 (XLSX 11 kb)

ESM 2 (XLSX 71 kb)

## Abbreviations

AFB1: aflatoxin B_1_
AFG1: aflatoxin G_1_
AFM1: aflatoxin M_1_
BEA: beauvericin
EU: European Union
FA: fusaric acid
FB1: fumonisin B_1_
FB2: fumonisin B_2_
LC-MS/MS: combined liquid chromatography-tandem mass spectrometry
LC-TOF-MS: combined liquid chromatography-time-of-flight mass spectrometry
Me-ST: O-methyl sterigmatocystin
NPA: 3-nitropropionic acid
OTA: ochratoxin A
ST: sterigmatocystin
TeA: tenuzonic acid
